# ETAPOD: A forecast model for prediction of black pod disease outbreak in Nigeria

**DOI:** 10.1371/journal.pone.0209306

**Published:** 2020-01-10

**Authors:** Peter M. Etaware, Abiodun R. Adedeji, Oyedeji I. Osowole, Adegboyega C. Odebode

**Affiliations:** 1 Department of Botany, Faculty of Science, University of Ibadan, Ibadan, Oyo State, Nigeria; 2 Cocoa Research Institute of Nigeria (CRIN), Idi-Ayunre, Ibadan, Oyo State, Nigeria; 3 Department of Statistics, Faculty of Science, University of Ibadan, Ibadan, Oyo State, Nigeria; George Mason University, UNITED STATES

## Abstract

Food poisoning and environmental pollution are products of excessive chemical usage in Agriculture. In Nigeria, cocoa farmers apply fungicides frequently to control black pod disease (BPD), this practice is life threatening and lethal to the environment. The development of a warning system to detect BPD outbreak can help minimize excessive usage of fungicide by farmers. 8 models (MRM_1_-MRM_8_) were developed and 5 (MRM_1_-MRM_5_) selected for optimization and performance check. MRM_5_ (ETAPOD) performed better than the other forecast models. ETAPOD had 100% performance rating for BPD prediction in Ekiti (2009, 2010, 2011 and 2015) with model efficiency of 95–100%. The performance of the model was rated 80% in 2010 and 2015 (Ondo) with model efficiency of 85–90%, 70% in 2011 (Osun) with model efficiency of 81–84%, 60% in 2010 (Ondo and Osun) and 2015 (Osun) with model efficiency of 75–80%, 40% in 2009 (Osun) with model efficiency of 65–69% and 0% 1n 2011 (Ondo) with model efficiency between 0 and 49%. ETAPOD is a simplified BPD detection device for the past, present and future.

## Introduction

Global warming, food poisoning and environmental pollution are current problems emanating from excessive exposure to and combustion of chemical substances. The management of BPD is a major challenge to cocoa farmers in Nigeria as they frequently apply fungicide to safeguard their crops without consideration for the safety of life and the environment [1]. BPD is more established in West Africa than in any other parts of the world [2]. Adegbola [2] in his review of Africa estimated the average occurrence of the disease as 40% in several parts of West Africa and up to 90% in certain places in Nigeria [3]. In Nigeria, cocoa export made over 80% GNI before the 1960s [3], it was reduced to 37.9% in 1997 [4] due to BPD infection and other factors, yet cocoa export remained more profitable than Rubber, Palm fruits, Groundnut, Yam, Cassava, Maize, Millet, and Sorghum [3]. Cocoa yield decline started in 1971–1972 (255,000 to 241,000Mt), through 1978–1979 (137,000Mt) to 1986 (58,700Mt), with an increase from 165,000–180,000Mt between 2000 and 2003 [5]; [6]. The increase in cocoa production was entirely due to the expansion in production area rather than increases in cocoa yield [7].

Global climate change is one of the major factors responsible for the inconsistent fluctuations in BPD outbreak experienced annually worldwide, due to its influence on the physiology of the pathogen(s), the suitability of the environment for microbial activities and the susceptibility of the host plant(s) to microbial attack [8]. The irregular rainfall pattern and inconsistent mode of BPD occurrence in Nigeria makes it nearly impossible to control it effectively. The efficacy of the existing management strategies (cultural, physical, biological and chemical control measures) are fast declining, as such increased fungicide dosages and frequent applications are methods devised by indigenous cocoa farmers to protect their crops from the disease. Hence, an urgent need for modern approach in the control of BPD in West Africa is imminent so as to reduce the level of exposure of cocoa pods to chemicals and also decrease the amount of chemical residues in the environment [9].

Plant disease forecasting (advance disease management strategy) advocates the use of plethora management techniques directed by a rational decision making system, such that indigenous cocoa farmers worldwide will be duly informed whenever BPD outbreak is suspected and the intensity of the outbreak quantified to avoid excessive use of preventive chemicals. This research seeks to develop a forecast model for BPD prediction so as to provide information on its outbreak and the areas suspected to be under severe invasion. In the nearest future, the quantity of preventive control measures required to combat the disease will be determined using simple computer algorithms in order to minimize fungicide misuse, reduce the level of chemical pollutants in the environment, increase cocoa productivity, and reduce the risk of chemical poisoning or deaths associated with consumption of toxic chemicals substances.

## Materials and methods

### The area of focus

**Fig 1:**
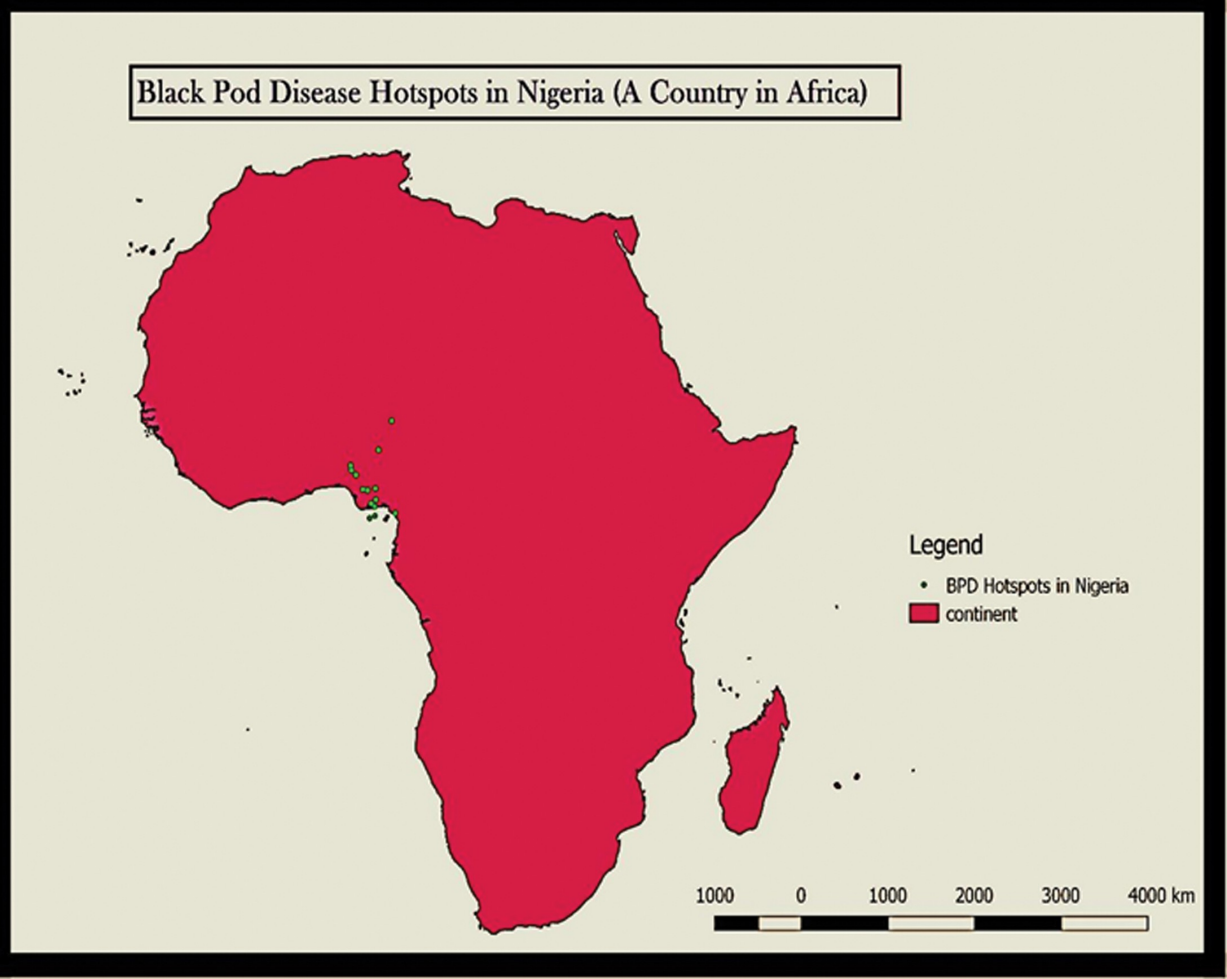
Black pod disease invasion hotspots in Nigeria (West Africa).

**Fig 2:**
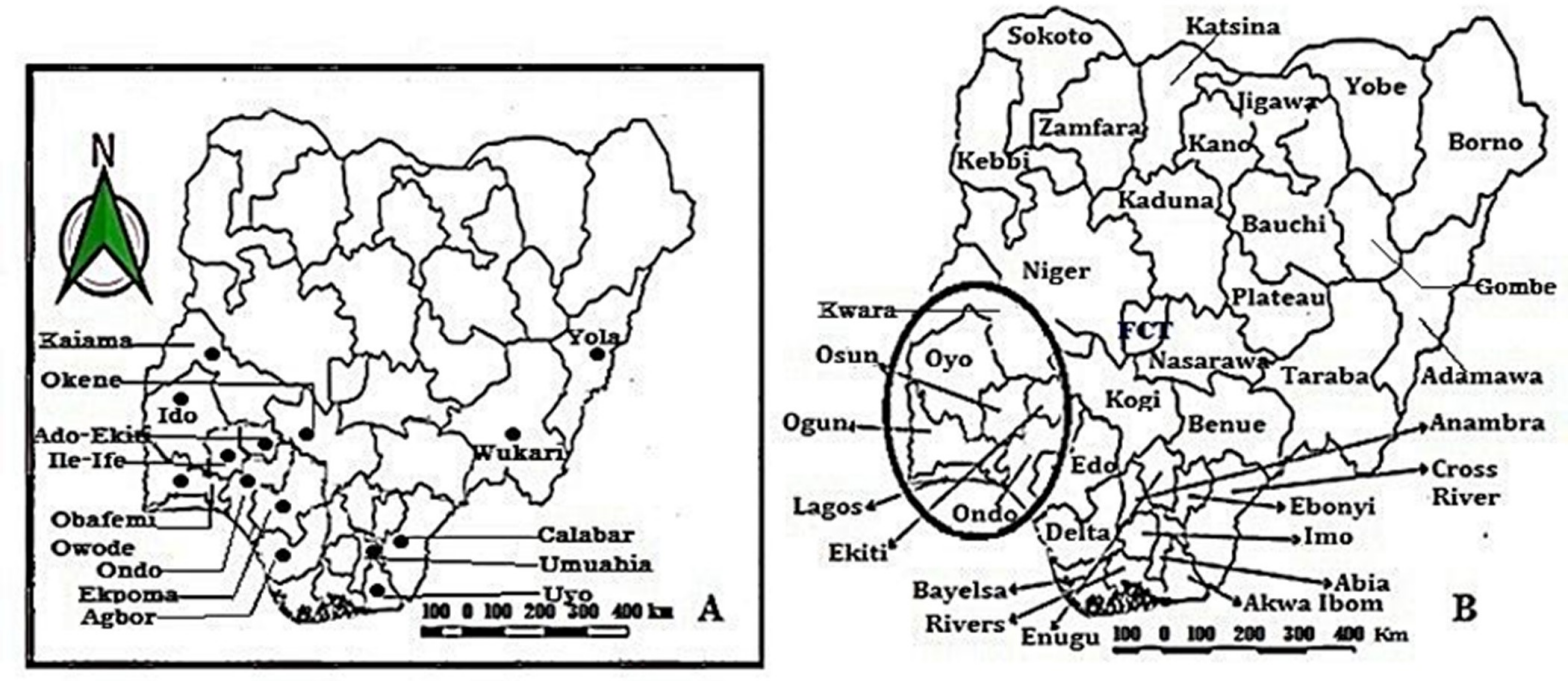
The map of Nigeria (A) Cocoa production sites (B) Southwestern Nigeria in circle.

**Fig 3:**
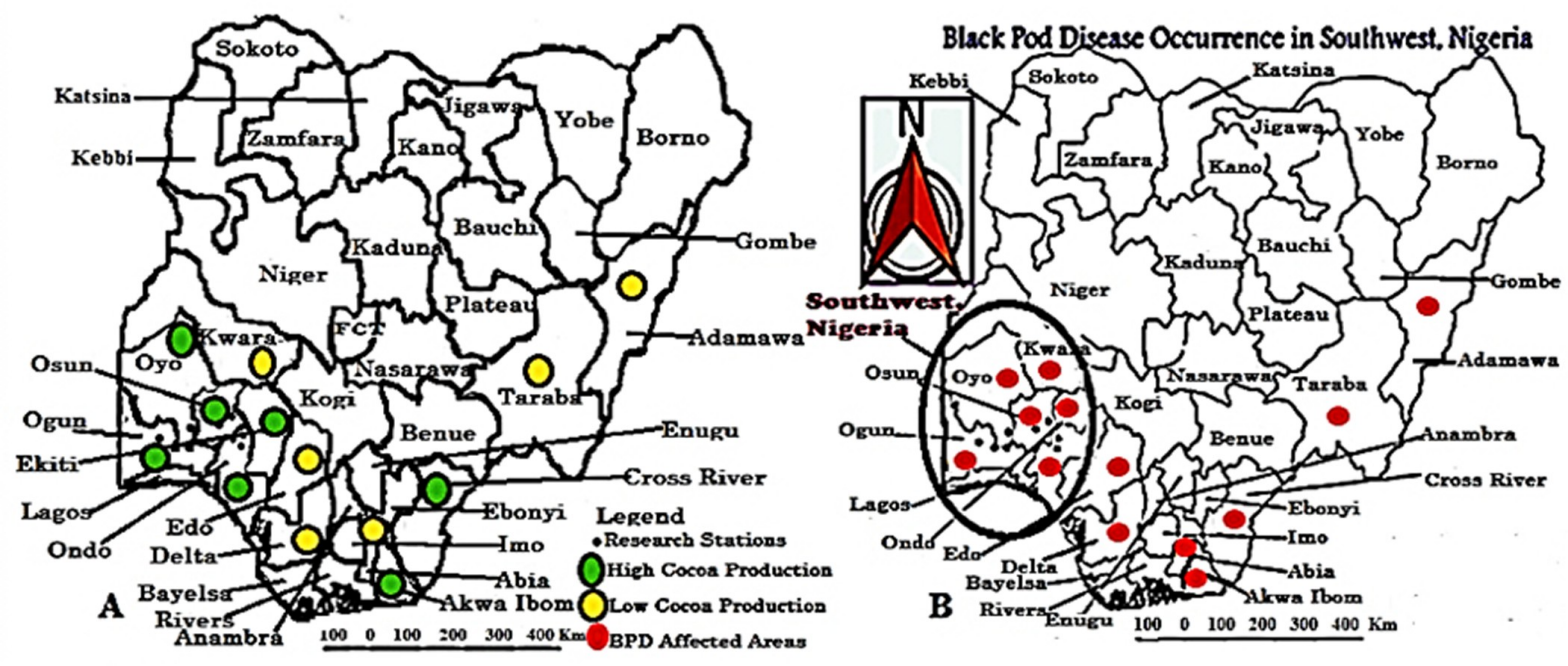
Cocoa production and BPD outbreak in (A) Nigeria (B) Southwest, Nigeria.

The area of research focus was the Western part of Africa with specific preference to Nigeria (Fig 1). The Southwestern region of Nigeria was used as a case study for result validation; this region was clearly described in Fig 2. The co-ordinates of the area of focus were determined using the blackberry mobile Global Positioning System (GPS) device (version 6.0) and a mobile satellite GPS receiver model GARMIN Etrex 10 obtained from the Department of Botany, Faculty of Science, University of Ibadan, Ibadan, Nigeria. Cocoa producing States in Nigeria were shown in Table 1 and Fig 3.

**Table 1:** Cocoa producing states in Nigeria and possible areas of BPD attack.

S/N	States	Area	Longitude (°E)	Latitude (°N)
1	Abia	Umuahia	07.48	05.52
2	Adamawa	Yola	12.47	09.23
3	Akwa-Ibom	Uyo	07.92	05.05
4	Cross River	Calabar	08.35	04.97
5	Delta	Agbor	06.15	06.25
6	Edo	Ekpoma	06.07	06.75
7	Ekiti	Ado-Ekiti	05.20	07.60
8	Kwara	Kaiama	03.95	09.61
9	Kogi	Okene	06.23	07.55
10	Ogun	Ọbáfémi-Owódé	03.50	06.95
11	Ondo	Ondo	04.83	07.10
12	Osun	Ile Ife	04.55	07.47
13	Oyo	Ido	03.71	07.50
14	Taraba	Wukari	09.78	07.88

### Black pod disease (BPD) data

Documented reports of BPD outbreaks within Southwestern Nigeria was obtained from Cocoa Research Institute of Nigeria (CRIN), Ìdí-Ayunrẹ, Ibadan, Oyo State, Nigeria and the report of Lawal and Emaku [10]. The total data collected spanned from 1985 to 2014. These served as secondary data.

### Meteorological data

Weather data from 1985 to 2016 within Southwest, Nigeria were also collected from the report of Lawal and Emaku [10], National Bureau of Statistics (NBS) Ibadan, Oyo State, the Meteorological Station of Cocoa Research Institute of Nigeria (CRIN), Ìdí-Ayunrẹ, Ibadan, Oyo State, Nigerian Meteorological Station (Nimet), and the Department of Geography, University of Ibadan, Ibadan, Oyo State, Nigeria. These were also classified as secondary data.

### Data analysis

Qualitative data were represented as charts and graphs plotted using SPSS, version 20.0 for 32 bits resolution, while the analysis of variance was carried out using COSTAT 6.451 software. The homogeneity of means was determined using Duncan Multiple Range Test (DMRT). The proposed forecast model(s) were templates of multiple regression equation(s) developed from the meteorological and BPD data (Secondary data). The models were designed using Minitab 16.0 software and programmed on Microsoft Excel Worksheet 2007 service pack for easy access. Model selection was aided by Pearson’s Product Moment of Correlation (PPMC) “R-Sq”, the Adjusted Coefficient of Correlation of the developed models (R-Sq_Adj._), the Standard Error of Regression (SER) and Root Mean Square Error of Prediction (RMSE_pred._). The Error of BPD prediction was determined using E = (Y-Ŷ)^2^.

### Model validation

The data used for confirmation of the predicted BPD outbreak by the developed forecast model was obtained from the research work of Oyekale [11], [12]. The template for validation (accuracy check) was stated in Table 2.

**Table 2:** Determination of the level of accuracy of the BPD model.

BPD outbreak			
Cocoa Trees Affected	BPD Prediction	Accuracy	BPD Prediction Capacity
100	100	100%	Best
100	75	75%	Good
100	50	50%	Poor
100	25	25%	Very Poor
100	0	0%	Bad

**© Etaware Peter Mudiaga** (2015/2016 Research)

## Results

The BPD function was developed using simple mathematical rule

Y=F(x)=ax+b

Where, a = Coefficient of x, x = independent variable, b = Constant, Y = Response variable and F = The Function of the variable x.

Thus,

BPD Outbreak=F(Host, Pathogen, Environment)=a(Host, Pathogen, Environment)+b

Mathematically,

Recall,Y=F(x)=ax+b

Then,D=F(H, P, E)=a(H, P, E)+b (1)

In any case the influence of man and vectors (Ants, Termites, and Rodents etc.) serve as constants in the equation because they influence the spread of BPD in the field, coupled with the timely combination of the key factors responsible for BPD development.

BPD Outbreak=F(Host, Pathogen, Environment)=a(Host, Pathogen, Environment)+(Time+Man+Vector)

Mathematically,

Recall,Y=F(x)=ax+b

Then, D=F(H, P, E)=a(H, P, E)+(T+M+V) (2)

Where, D=BPD Outbreak, H=Host, P=Pathogen, T=Time, M=man, V=Vectors, E=Environment, x=(H, P, E),and b=(T+M+V)

Therefore, the equation can be written as the first order differential equation for BPD outbreak.

δD_=δD_(HPE)+δD_(HPE)+δD_(HPE)δT δH δP δE (3)

A forecast system for prediction of any plant disease can be developed from any of these:

1. The study of the life cycle of the Host Plant (i.e. sowing date, flowering, fruiting etc.)
  δD_=δD_(HPE)δT δH (4)
2. The Pathogen’s Ecology (inoculum load, spore, toxin or enzyme production, life cycle etc.)
  δD_=δD_(HPE)δT δP (5)
3. The study of surrounding Environmental Factors (Rainfall, Temperature, soil moisture etc.)
  δD_=δD_(HPE)δT δE (6)

The forecast models were structured using the Multiple Regression Equation (MRM):

$$\begin{array}{l} \text{Y}=\beta_{0}+\beta_{1}{\text{X}}_{1}+\beta_{2}{\text{X}}_{2}+\beta_{3}{\text{X}}_{3}+\beta_{4}{\text{X}}_{4}+\beta_{5}{\text{X}}_{5}\ldots \ldots \ldots \ldots \ldots \ldots \ldots \ldots \ldots \ldots \ldots +\beta_{\text{n}}{\text{X}}_{\text{n}}+£\\ \text{Since}\;\alpha =\beta_{0,}\text{Y}=\alpha +\beta_{1}{\text{X}}_{1}+\beta_{2}{\text{X}}_{2}+\beta_{3}{\text{X}}_{3}+\beta_{4}{\text{X}}_{4}+\beta_{5}{\text{X}}_{5}\ldots \ldots \ldots \ldots \ldots \ldots ..+\beta_{\text{n}}{\text{X}}_{\text{n}}+£ \end{array}$$

Where, Y = Response variable, X_1_, X_2_, X_3_, X_4_, X_5_,…..X_n_ = Predictors, β_1_, β_2_, β_3_, β_4_, β_5_……β_n_ = The slopes, α = General constant and £ = The error factor for the predictors [13].

Therefore, the development of BPD forecast system for cocoa required an equation encompassing all the predictors necessary for the disease development. An example of such model was given thus:

$$\text{Y}=\alpha +\beta_{1}\left(\text{Disease Incidence}\right)+\beta_{2}\left(\text{Disease Severity}\right)+\beta_{3}\left(\text{Inoculum Size}\right)+\beta_{4}\left(\text{Rainfall}\right)+\beta_{5}\left(\text{Temperature}\right)+\beta_{6}\left(\text{Humidity}\right)+\beta_{7}\left(\text{Sunlight Duration}\right)+\beta_{8}\left(\text{Wind Speed}\right)+\beta_{9}\left(\text{Time}\right)+\beta_{10}\left(\text{Pressure}\right)+£$$

Or

$$\text{Y}=\beta_{0}+\beta_{1}\left(\text{Disease Incidence}\right)+\beta_{2}\left(\text{Disease Severity}\right)+\beta_{3}\left(\text{Inoculum Size}\right)+\beta_{4}\left(\text{Rainfall}\right)+\beta_{5}\left(\text{Temperature}\right)+\beta_{6}\left(\text{Humidity}\right)+\beta_{7}\left(\text{Sunlight Duration}\right)+\beta_{8}\left(\text{Wind Speed}\right)+\beta_{9}\left(\text{Time}\right)+\beta_{10}\left(\text{Pressure}\right)+£$$

In any case the individual predictors were tested against the response variable to ascertain their role(s) in black pod disease outbreak.

**Fig 4:**
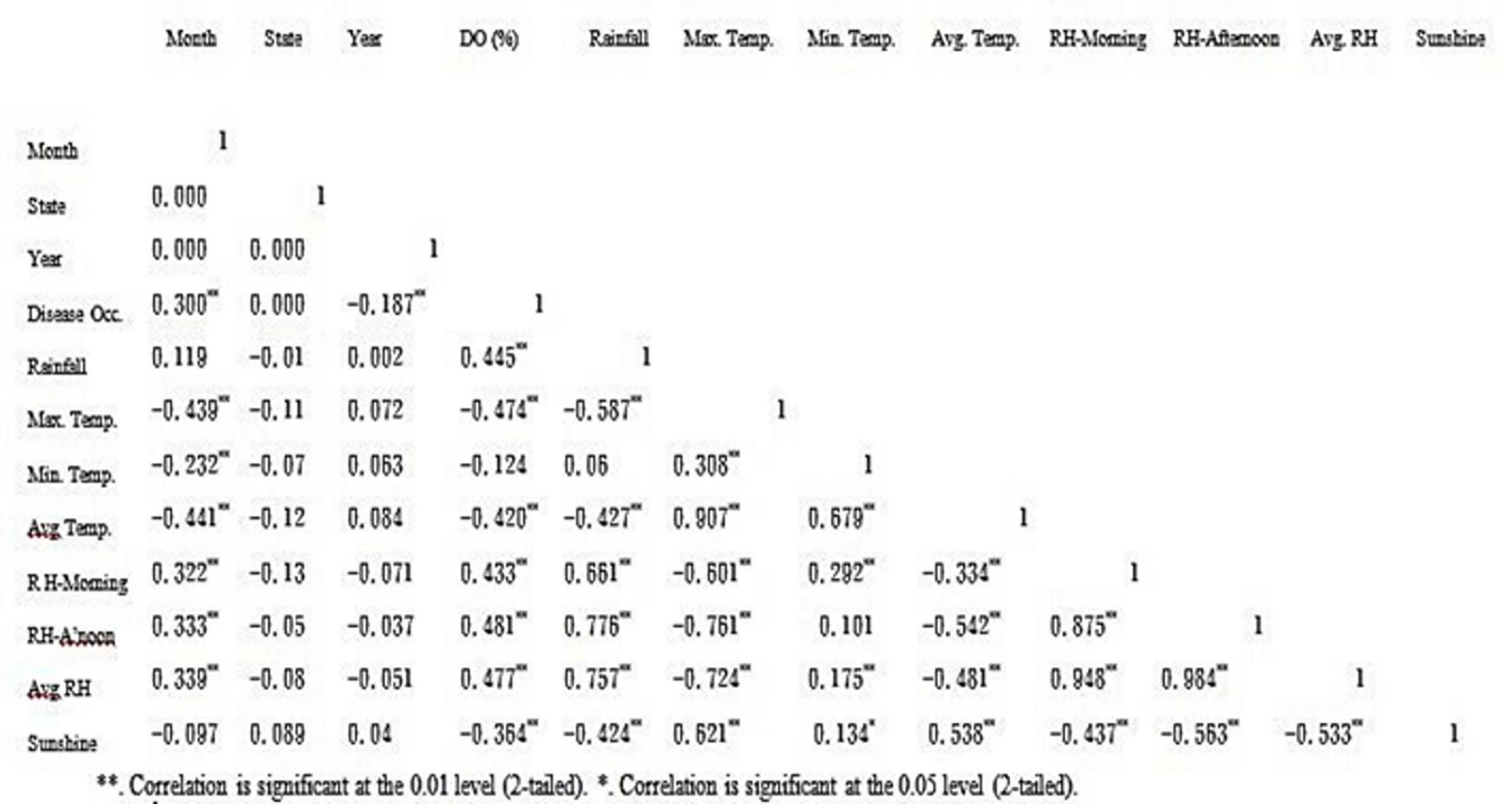
Relationship between BPD outbreak and climatic factors in Southwest, Nigeria.

**Fig 5:**
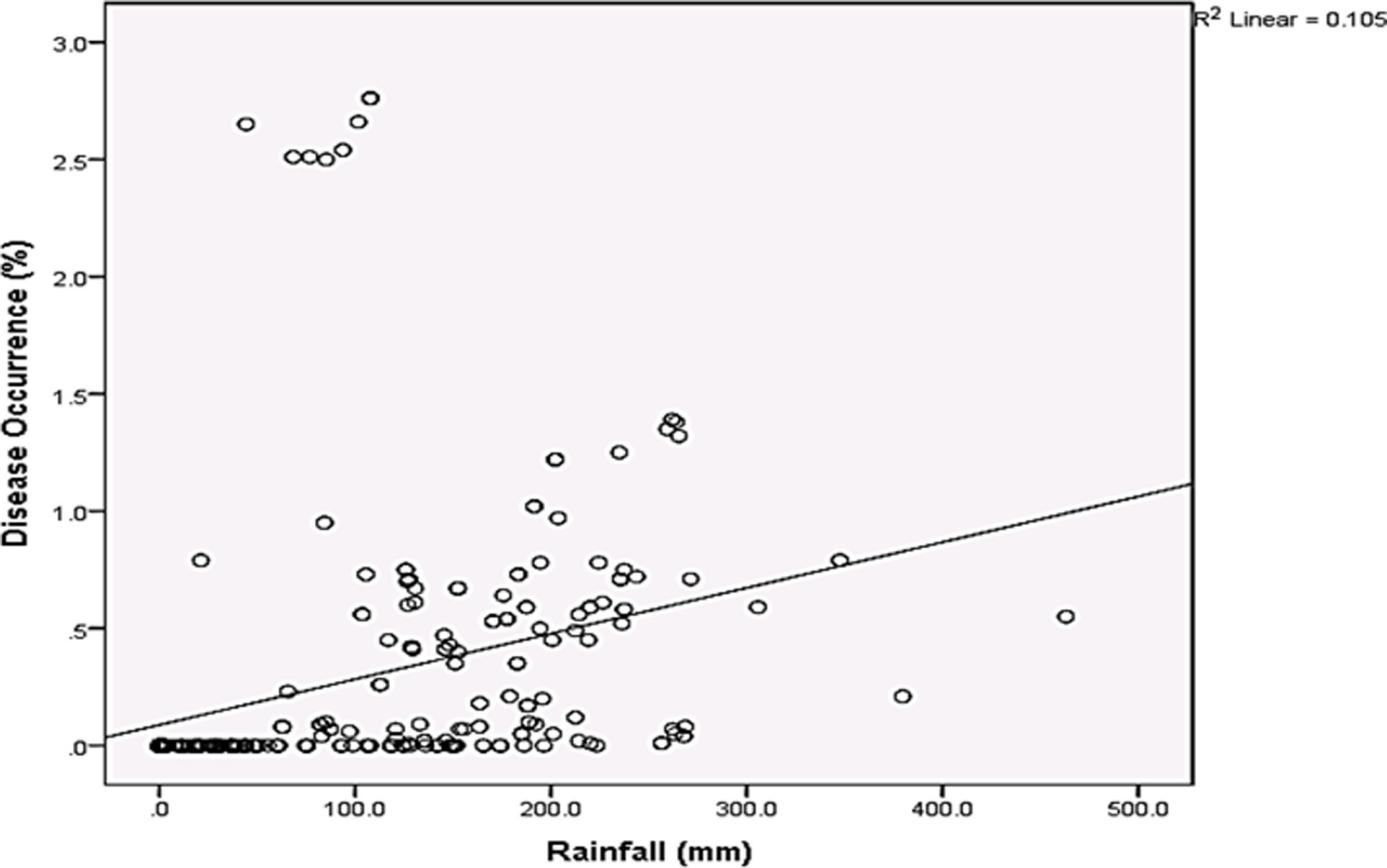
BPD outbreak and rainfall (1991–1995).

**Fig 6:**
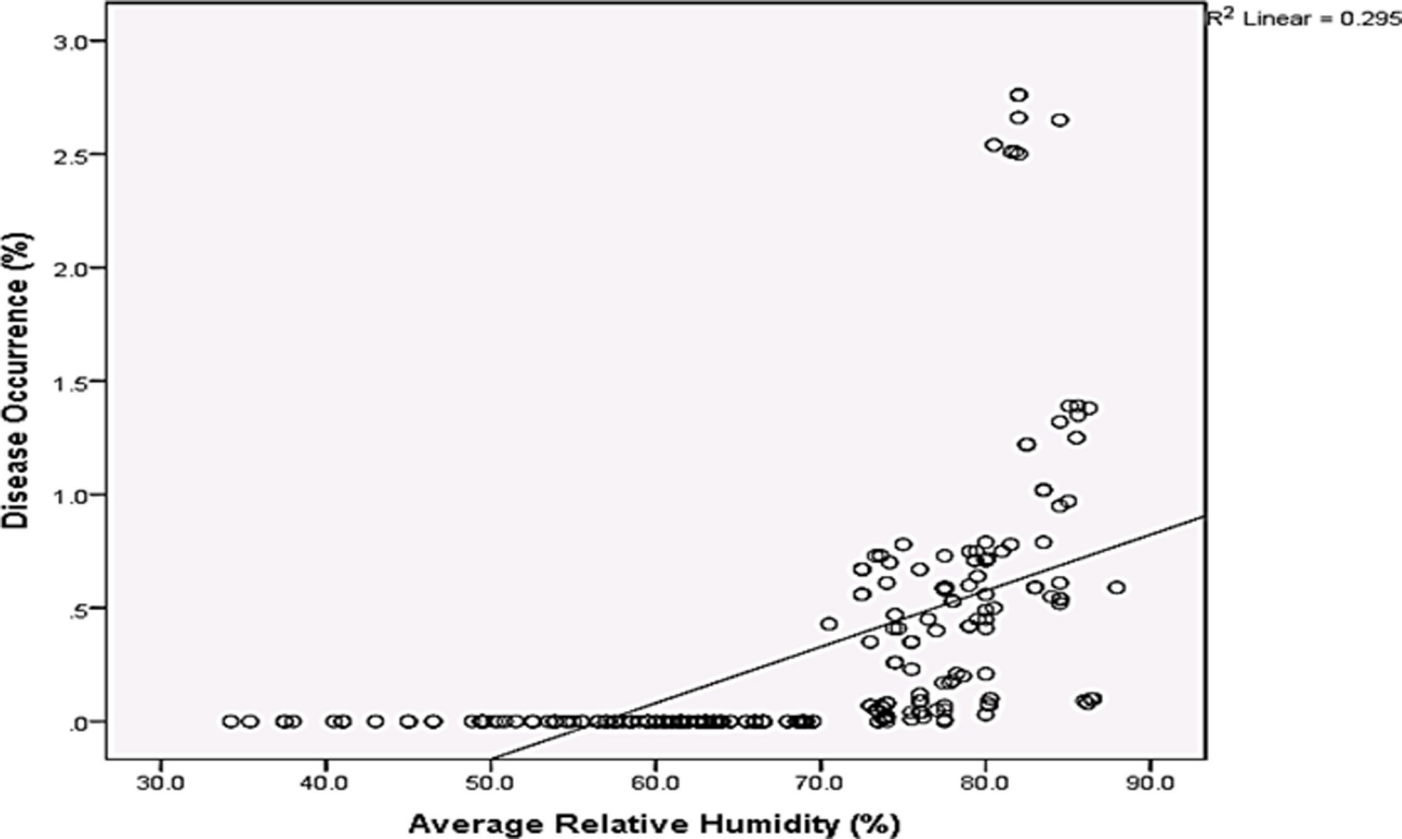
BPD outbreak and average relative humidity in Southwestern Nigeria (1991–1995).

**Fig 7:**
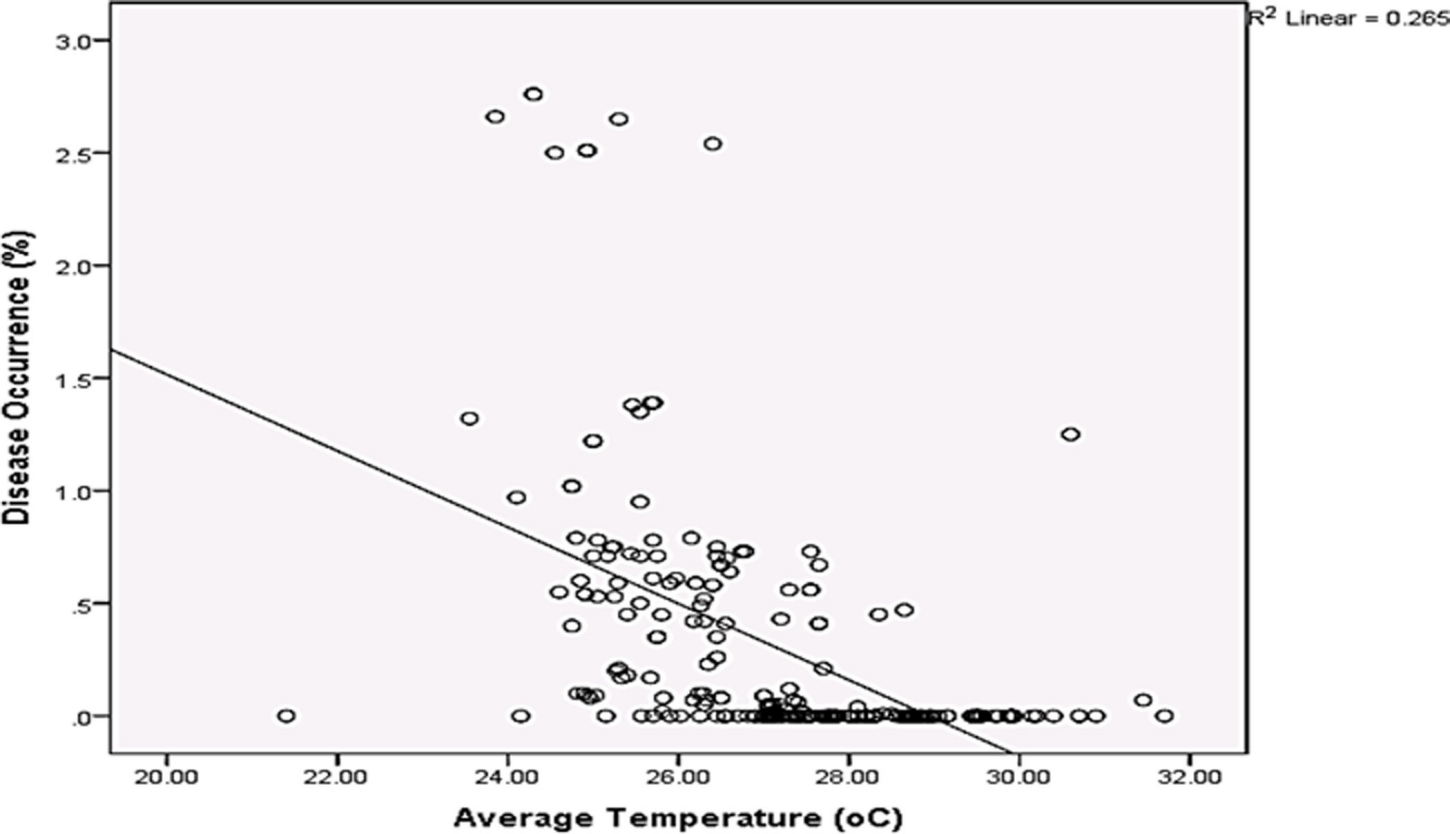
BPD outbreak and average temperature in Southwestern Nigeria (1991–1995).

**Fig 8:**
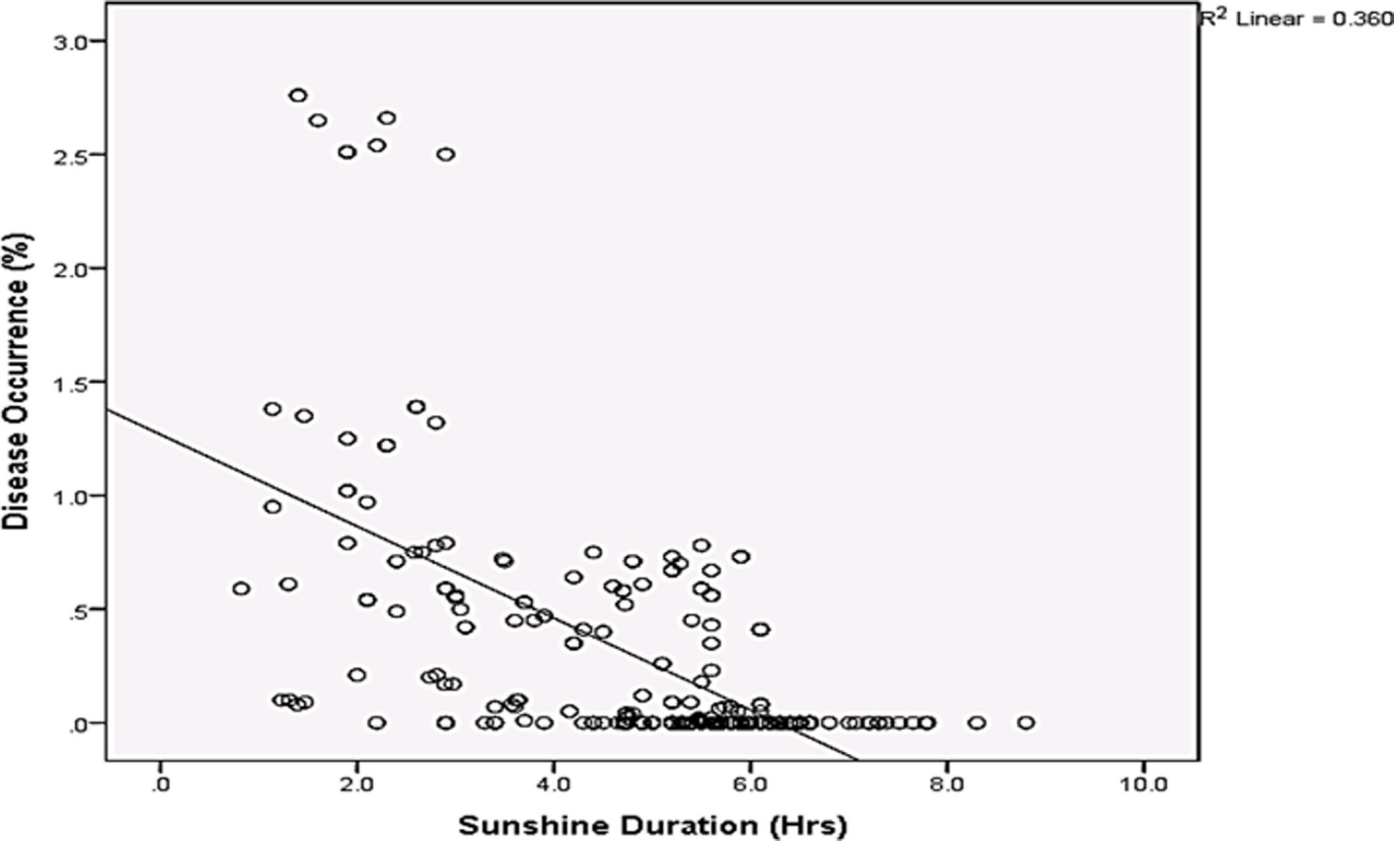
BPD outbreak and sunshine duration (1991–1995).

**Fig 9:**
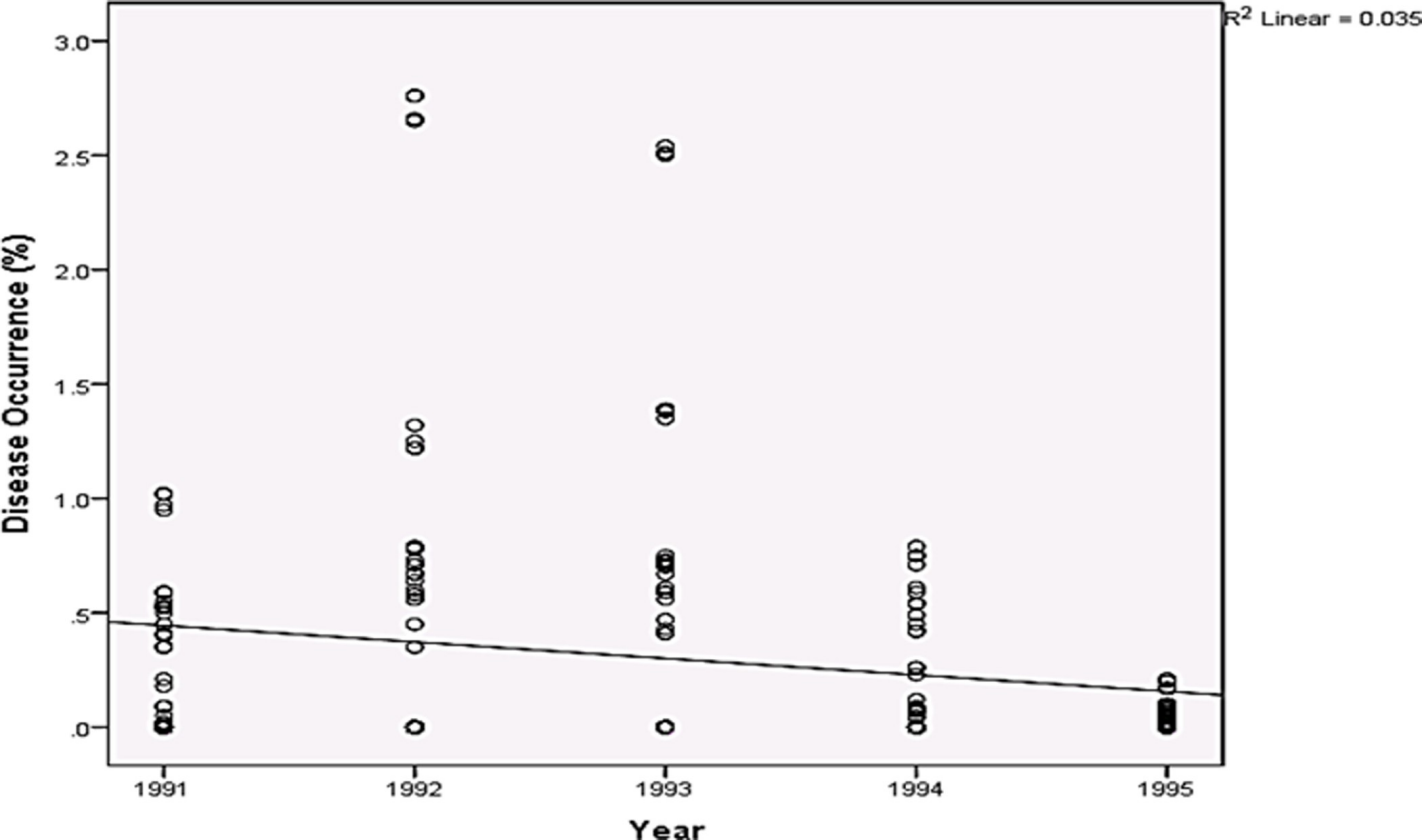
BPD occurrence and the years of BPD documentation (1991–1995).

**Fig 10:**
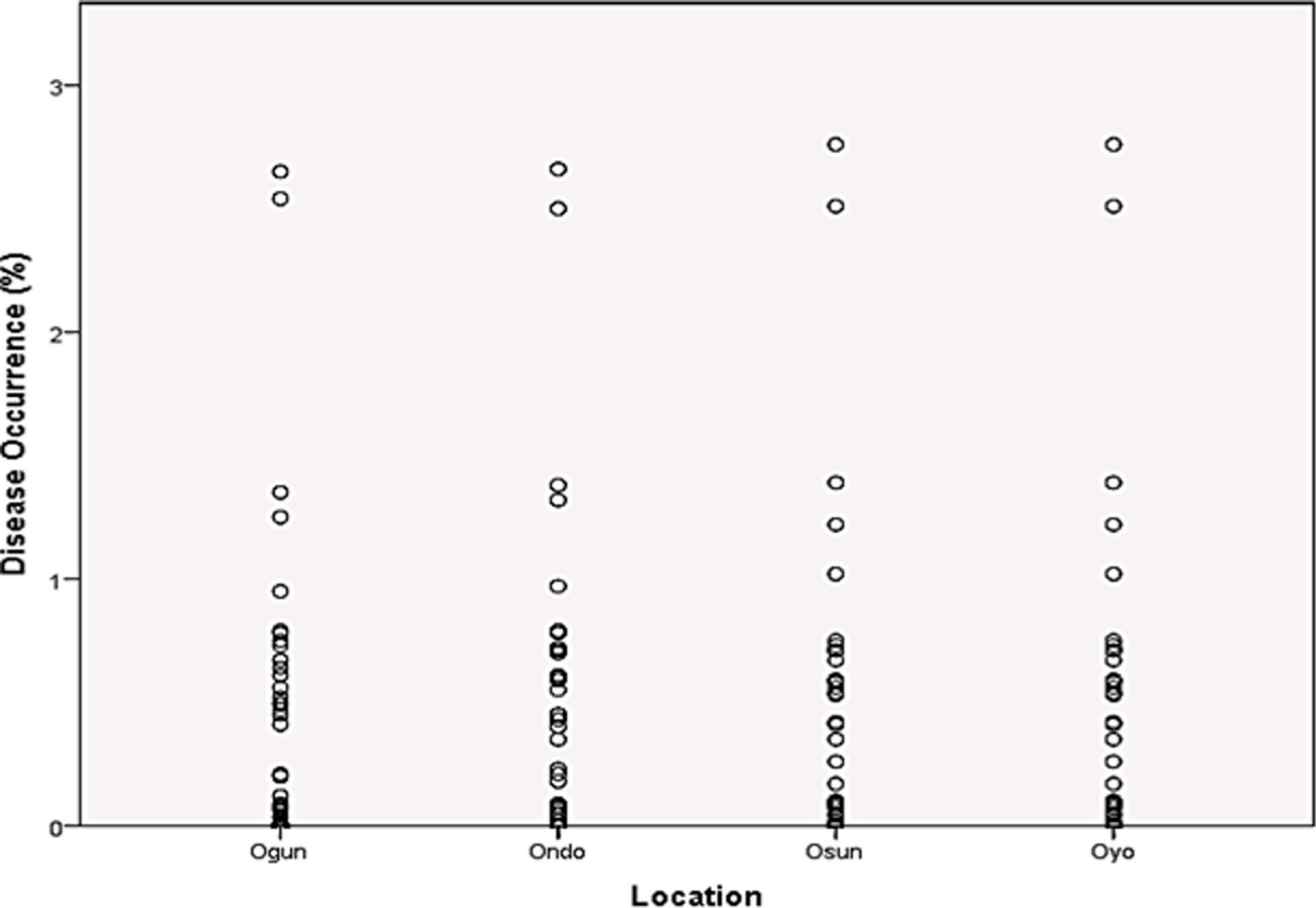
BPD occurrence and sample station location (1991–1995).

**Fig 11:**
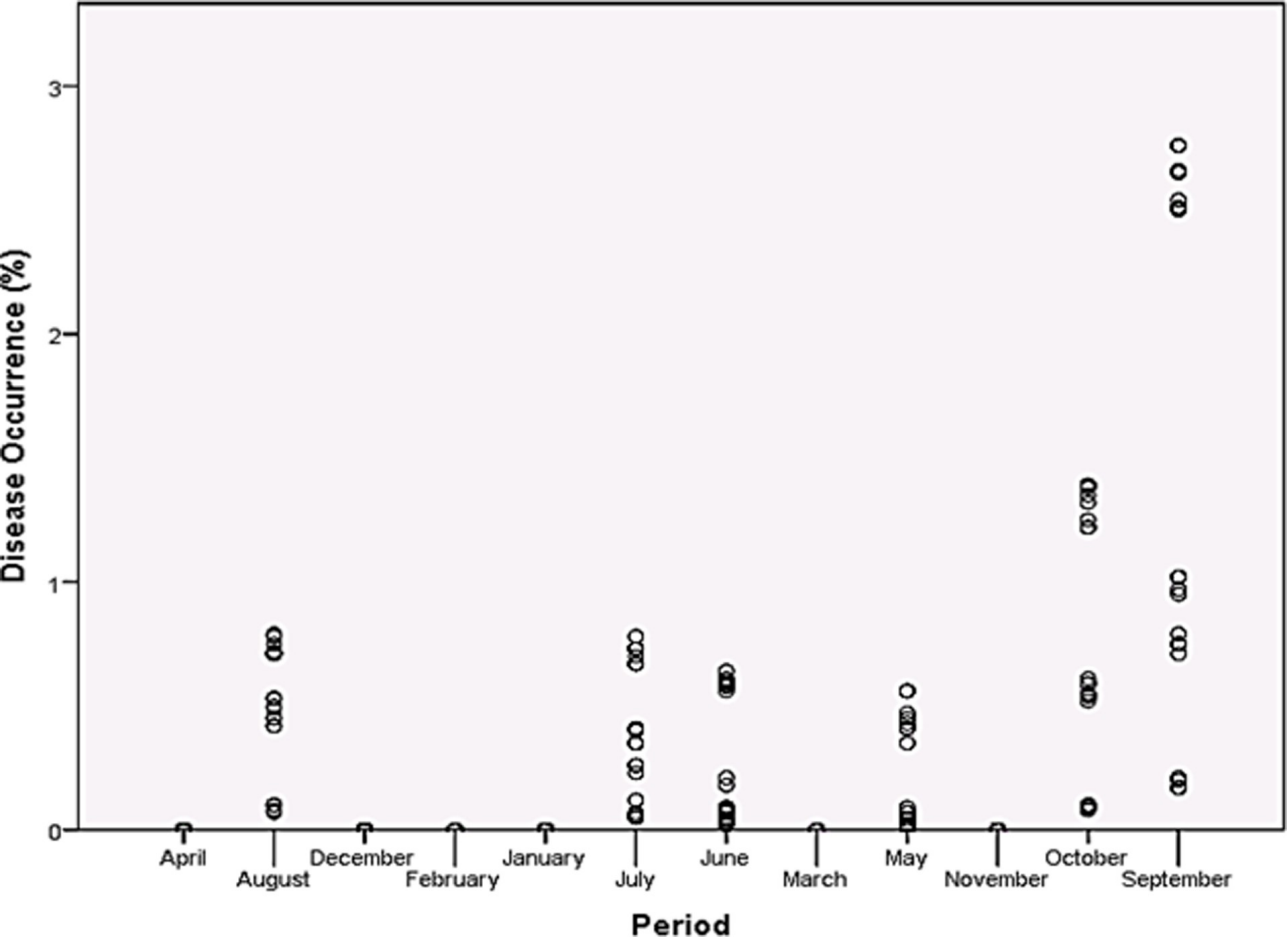
BPD outbreak and period (in months) of observation (1991–1995).

Rainfall and average relative humidity had a positive correlation with BPD outbreak i.e. r = 0.445 and 0.477 (Fig 4), and r^2^ = 0.105 (Fig 5) and 0.295 (Fig 6), respectively. The average temperature, sunshine duration and the year of observation had negative association with BPD outbreak in Southwest, Nigeria (r = -0.420, -0.364 and -0.018 (Fig 4), and r^2^ = 0.265 (Fig 7), 0.360 (Fig 8) and 0.035 (Fig 9), respectively). It was however observed that there was no relationship between the locations of cocoa farms (Fig 10), the specific period (month) when the disease was observed (Fig 11) and BPD outbreak in Nigeria.

### The weather pattern of Southwest, Nigeria and how it affects BPD development

**Fig 12:**
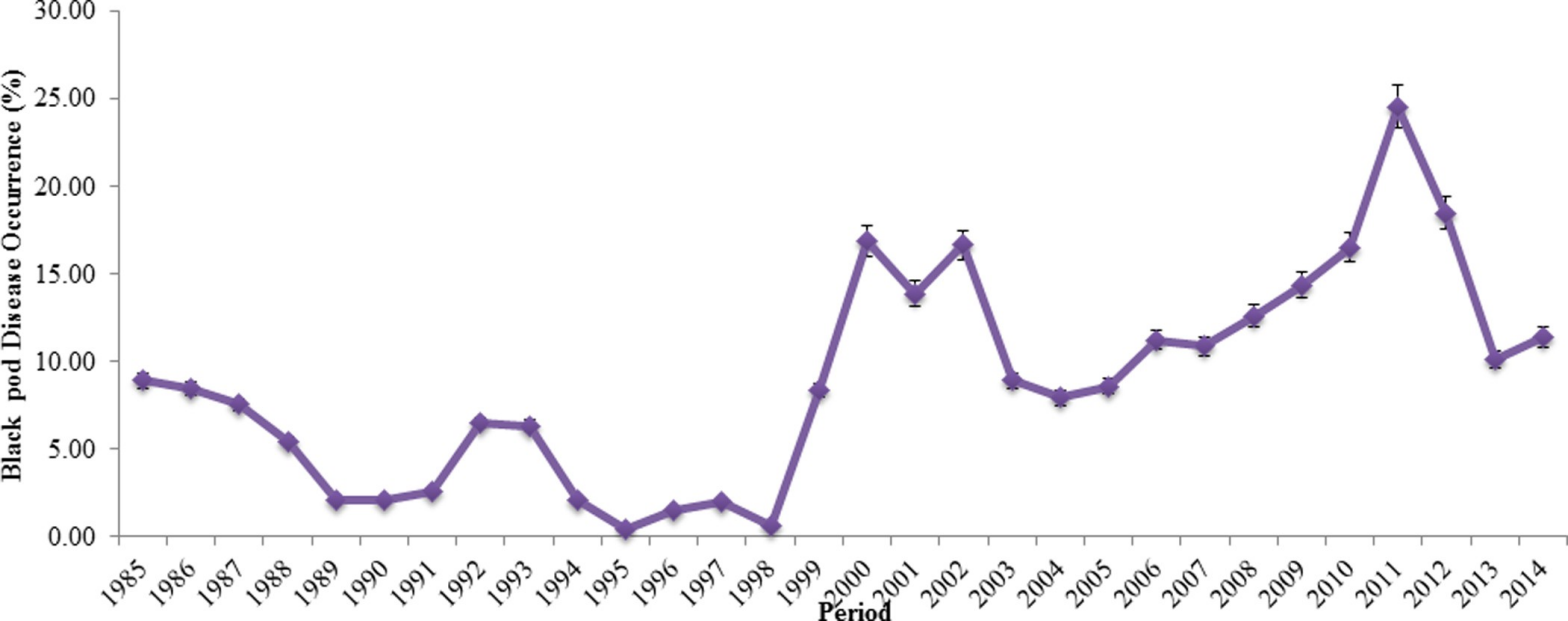
Records of BPD outbreak in Southwest, Nigeria.

The weather pattern for Southwest, Nigeria in the late 1900s (20^th^ Century) showed that the height of rainfall across the four (4) States investigated was between the months of March and October from 1991 to 1995, suggesting the possibility of infection within these periods (Table 3). *Phytophthora megakarya* thrives better between 20°C and 30°C, therefore the specific periods of the year that favoured such temperature values in Ogun, Ondo, Osun and Oyo States were June, July, August, and September in 1991 to 1995 (Table 4). On the Contrary, the minimum temperature all year round favoured the proliferation of the pathogen (Table 5). A relative humidity value of 75% and above favoured the establishment of BPD, therefore, periods of the year that had high relative humidity were March through October from the early morning readings taken 1991 to 1995, suggesting the possibility of infection within these periods also (Table 6). Judging by the trend of afternoon readings the periods of June through September across all the years favoured BPD proliferation (Table 7). These periods possibly served as an interlude for proliferation and spread of the pathogen leading to possible infection of predisposed cocoa plants judging from the BPD occurrence report given by the Cocoa Research Institute of Nigeria (CRIN) from 1985–2014 as shown in Fig 12.

### Black pod disease trend in Southwest, Nigeria

Fig 12 showed a decrease in BPD occurrence in Southwest, Nigeria from 8.93% in 1985 to 2.60% in 1991, it later increased in 1992 (6.51%) with constant fluctuation to 1999 (8.35%). BPD outbreaks was massive in 2000 (16.90%), 2001 (13.90%), 2002 (16.67%), through to 2006 (11.25%). Also, a combination of a low temperature (23.4–32.4°C), high relative humidity (70–100%) and heavy rainfall (1036.9–1604.4mm) resulted in massive BPD occurrence as shown in 1985–1987, and 1999–2014 (Fig 12).

**Table 3:** Monthly rainfall distribution for the Southwest, states of Nigeria.

		Rainfall (mm)/Months											
State	Year	Jan	Feb	Mar	April	May	June	July	Aug	Sept	Oct	Nov	Dec
**Ogun**	1991	2.50	60.0	38.1	118.1	127.1	179	236.2	84.3	194.4	129.4	0.00	4.00
	1992	0.00	0.00	8.40	149.7	116.9	175.7	235	44.3	224.3	105.5	20.7	TR
	1993	NA	NA	NA	NA	NA	NA	NA	NA	NA	NA	NA	NA
	1994	12.1	1.6	124.1	60.2	82.9	120.7	130.5	21.2	212.5	212.5	15.7	0.00
	1995	0.00	4.00	150.6	124.8	220.1	120.8	133	195.7	163.5	97.1	NA	NA
**Ondo**	1991	1.20	98.6	136	223.2	201.2	163.7	463	203.6	200.7	152.6	TR	10.4
	1992	0.00	0.00	40.8	107.8	151.1	127	265.3	101.7	347.6	194.6	25.6	0.00
	1993	NA	NA	NA	NA	NA	NA	NA	NA	NA	NA	NA	NA
	1994	31.3	50.9	74.5	186.2	192.2	263.3	305.7	271.4	219.1	65.7	39.2	0.00
	1995	0.00	28.4	128	196.3	146.4	214.2	268.6	379.6	262.3	87.3	14.2	0.00
**Oyo**	1991	TR	165.5	19.0	174.1	135.3	82.3	219.9	191.4	170.4	182.8	2.2	26.4
	1992	0.00	0.00	28.5	92.9	103.6	237.4	202.3	107.8	127.4	152.5	36.2	0.00
	1993	0.00	NA	141.7	44	145.9	187.5	262	NA	235.5	183.2	NA	48.3
	1994	2.10	30.2	20.7	75.4	NA	62.9	177.4	125.9	128.8	112.7	17.6	0.00
	1995	0.00	11.4	106.3	118.5	256.6	267.8	188.9	188.1	84.9	185.1	36.6	TR
**Osun**	1991	TR	165.5	19.0	174.1	135.3	82.3	219.9	191.4	170.4	182.8	2.2	26.4
	1992	0.00	0.00	28.5	92.9	103.6	237.4	202.3	107.8	127.4	152.5	36.2	0.00
	1993	0.00	NA	141.7	44.0	145.9	187.5	262	NA	235.5	183.2	NA	48.3
	1994	2.10	30.2	20.7	75.4	NA	62.9	177.4	125.9	128.8	112.7	17.6	0.00
	1995	0.00	11.4	106.3	118.5	256.6	267.8	188.9	188.1	84.9	185.1	36.6	TR

**Data Source:** National Bureau of Statistics (NBS). TR—Trace quantity, NA—Not Available.

**Table 4:** Mean monthly maximum temperature reading for the Southwest, states of Nigeria.

		Max. Temperature (°C)/Months											
State	Year	Jan	Feb	Mar	April	May	June	July	Aug	Sept	Oct	Nov	Dec
**Ogun**	1991	34.3	37.4	35.6	33.7	32.6	31.6	29.5	28.4	30.1	30.5	33.6	33.6
	1992	34.5	37.3	36.3	35	32.7	30.3	38.3	28	29.2	31.9	33.2	34.8
	1993	35.1	35.8	34.6	35	33.1	31.1	NA	29.7	30.8	32	33.6	33.8
	1994	34	36.3	35.6	34.3	32.5	31.7	28.5	29.3	30.4	31.7	33.9	35.2
	1995	35.5	NA	NA	NA	33.1	31.2	NA	NA	NA	31.5	NA	NA
**Ondo**	1991	32.2	33.4	32.6	31.3	30.7	29.6	28.2	26.9	28.8	29.3	31.8	32.1
	1992	33	36	33.8	32.5	31.1	29	27	26.9	27.9	30	31.2	23.8
	1993	NA	NA	NA	NA	NA	NA	NA	NA	NA	NA	NA	NA
	1994	32	34.1	33.9	32.5	26.8	29	NA	NA	29	30.4	32.7	
	1995	NA	NA	NA	NA	NA	NA	NA	NA	NA	NA	NA	NA
**Oyo**	1991	33.5	34.9	34.6	33	31.6	31	29.3	27.7	28.1	30	32.2	32
	1992	32.9	36.2	35.5	33.7	31.8	29.9	28	27.2	28.3	30.9	32.1	33.3
	1993	33.1	34.6	33.5	33.1	32	30	NA	28.1	29.7	NA	31.9	NA
	1994	32.7	34.9	35.5	34	32	30.7	27.9	NA	30	30.7	33.2	33.8
	1995	NA	NA	NA	NA	NA	NA	NA	NA	NA	NA	NA	NA
**Osun**	1991	33.5	34.9	34.6	33	31.6	31	29.3	27.7	28.1	30	32.2	32
	1992	32.9	36.2	35.5	33.7	31.8	29.9	28	27.2	28.3	30.9	32.1	33.3
	1993	33.1	34.6	33.5	33.1	32	30	NA	28.1	29.7	NA	31.9	NA
	1994	32.7	34.9	35.5	34	32	30.7	27.9	NA	30	30.7	33.2	33.8
	1995	NA	NA	NA	NA	NA	NA	NA	NA	NA	NA	NA	NA

**Data Source:** National Bureau of Statistics (NBS). TR—Trace quantity, NA—Not Available.

**Table 5:** Mean monthly minimum temperature reading for the Southwest, states of Nigeria.

		Minimum Temperature (°C)/Months											
State	Year	Jan	Feb	Mar	April	May	June	July	Aug	Sept	Oct	Nov	Dec
**Ogun**	1991	23.8	26	25.2	23.7	24.2	23.8	23.1	22.7	22.8	22.6	24.2	22.5
	1992	20.5	24.1	25.5	23.3	24	22.9	22.9	22.6	22.4	23.2	22.3	23.2
	1993	21.1	24.5	23.7	24.5	24.2	23.5	NA	NA	22.8	23.3	23.8	22.2
	1994	23.1	25.1	24.8	25.1	23.7	31.2	22.9	23	23.2	22.9	22.5	20.2
	1995	22.2	NA	NA	NA	23.9	23.3	NA	NA	NA	23.3	NA	NA
**Ondo**	1991	19.6	22.6	22.7	21.2	21.9	21.2	21	21.3	21	20.2	21.1	18.2
	1992	15.3	18.8	22.8	22.9	21.8	20.7	20.1	20.8	20.9	21.4	20.2	19
	1993	17.3	20.6	21.9	23.1	22.9	22	NA	21.7	22.1	NA	22.1	NA
	1994	NA	NA	NA	NA	NA	NA	NA	NA	NA	NA	NA	NA
	1995	19.6	21.7	22.9	22.4	NA	21.2	NA	NA	21.7	21.2	20.7	NA
**Oyo**	1991	22.9	24.0	24.4	23.2	23.3	23	22.5	21.8	22.0	21.5	23.3	21.8
	1992	20.2	22.9	24.3	23.8	23.3	22.9	22	21.4	21.7	22.1	21.9	22.4
	1993	20.9	NA	22.9	23.5	23.3	22.4	22	NA	21.8	22.3	NA	22.2
	1994	22.4	24.1	24.3	23.9	22.7	22.3	21.9	NA	22.6	22.2	22.4	20.4
	1995	NA	NA	NA	NA	NA	NA	NA	NA	NA	NA	NA	NA
**Osun**	1991	22.9	24.0	24.4	23.2	23.3	23	22.5	21.8	22.0	21.5	23.3	21.8
	1992	20.2	22.9	24.3	23.8	23.3	22.9	22	21.4	21.7	22.1	21.9	22.4
	1993	20.9	NA	22.9	23.5	23.3	22.4	22	NA	21.8	22.3	NA	22.2
	1994	22.4	24.1	24.3	23.9	22.7	22.3	21.9	NA	22.6	22.2	22.4	20.4
	1995	NA	NA	NA	NA	NA	NA	NA	NA	NA	NA	NA	NA

**Data Source:** National Bureau of Statistics (NBS). TR—Trace quantity, NA—Not Available.

**Table 6:** Relative humidity values for the Southwest, states of Nigeria.

		**Relative Humidity in the morning at 9.00GMT (%)/Months**											
State	Year	Jan	Feb	Mar	April	May	June	July	Aug	Sept	Oct	Nov	Dec
**Ogun**	1991	81	64	81	84	83	87	90	89	87	87	84	78
	1992	55	70	76	81	83	85	89	89	88	86	76	78
	1993	53	80	77	78	82	85	NA	87	87	85	89	78
	1994	73	78	80	79	83	84	88	87	86	85	80	64
	1995	87	NA	NA	NA	82	85	NA	NA	NA	NA	85	NA
**Ondo**	1991	75	79	81	83	84	85	89	89	86	84	78	67
	1992	48	54	75	79	82	84	88	87	89	83	72	69
	1993	48	68	71	76	77	82	NA	NA	NA	NA	NA	NA
	1994	69	72	76	79	NA	NA	NA	85	85	82	71	NA
	1995	NA	NA	NA	NA	NA	NA	NA	NA	NA	NA	NA	NA
**Oyo**	1991	70	78	76	81	81	83	88	88	85	84	79	70
	1992	50	63	73	78	80	84	88	87	87	82	73	75
	1993	48	NA	75	78	80	83	86	NA	85	83	NA	73
	1994	68	73	74	75	82	81	89	NA	86	83	74	57
	1995	NA	NA	NA	NA	NA	NA	NA	NA	NA	NA	NA	NA
**Osun**	1991	70	78	76	81	81	83	88	88	85	84	79	70
	1992	50	63	73	78	80	84	88	87	87	82	73	75
	1993	48	NA	75	78	80	83	86	NA	85	83	NA	73
	1994	68	73	74	75	82	81	89	NA	86	83	74	57
	1995	NA	NA	NA	NA	NA	NA	NA	NA	NA	NA	NA	NA

**Data Source:** National Bureau of Statistics (NBS). TR—Trace quantity, NA—Not Available.

**Table 7:** Relative humidity values for the Southwest of Nigeria.

		Relative Humidity in the afternoon at 15.00GMT											
State	Year	Jan	Feb	Mar	April	May	June	July	Aug	Sept	Oct	Nov	Dec
**Ogun**	1991	48	54	38	63	72	73	79	80	74	73	35	47
	1992	31	31	47	57	70	74	82	80	75	69	56	45
	1993	28	41	50	58	67	75		74	75	67	59	48
	1994	46	37	52	59	68	71	81	73	74	67	52	35
	1995	35	NA	NA	NA	69	75	NA	NA	NA	NA	70	NA
**Ondo**	1991	44	50	55	64	71	71	79	81	73	70	53	40
	1992	27	21	45	60	64	74	81	77	78	67	52	41
	1993	28	35	48	55	64	66	NA	NA	NA	NA	NA	NA
	1994	45	37	50	58	NA	NA	NA	75	75	69	53	NA
	1995	NA	NA	NA	NA	NA	NA	NA	NA	NA	NA	NA	NA
**Oyo**	1991	43	49	50	58	67	69	78	79	71	67	54	45
	1992	32	27	44	55	65	71	77	77	73	63	55	46
	1993	NA	NA	NA	NA	NA	NA	NA	NA	NA	NA	NA	NA
	1994	46	38	46	57	64	67	80	NA	72	66	49	36
	1995	NA	NA	NA	NA	NA	NA	NA	NA	NA	NA	NA	NA
**Osun**	1991	43	49	50	58	67	69	78	79	71	67	54	45
	1992	32	27	44	55	65	71	77	77	73	63	55	46
	1993	NA	NA	NA	NA	NA	NA	NA	NA	NA	NA	NA	NA
	1994	46	38	46	57	64	67	80	NA	72	66	49	36
	1995	NA	NA	NA	NA	NA	NA	NA	NA	NA	NA	NA	NA

**Data Source:** National Bureau of Statistics (NBS). TR—Trace quantity, NA—Not Available.

### Development of prediction models for black pod disease in Nigeria

Several models were developed to predict BPD outbreak in Southwest, Nigeria.

### Model 1 (MRM_1_)

General Equation (1991–1995)

$${\text{Y}=\alpha +\beta }_{1}{\text{X}}_{1}{+\beta }_{2}{\text{X}}_{2}{–\beta }_{3}{\text{X}}_{3}{+\beta }_{4}{\text{X}}_{4}{–\beta }_{5}{\text{X}}_{5}{–\beta }_{6}{\text{X}}_{6}{+\beta }_{7}{\text{X}}_{7}{–\beta }_{8}{\text{X}}_{8}{–\beta }_{9}{\text{X}}_{9}$$

BPD Outbreak(%)=124.8+0.03(Month)+0.01(State)-0.06(Year)+0.002(Rainfall)-0.003(Max. Temperature)-0.04(Min. Temperature)+0.01(Relative Humidity[Morning])-0.0003(Relative Humidity[Afternoon])-0.05(Sunshine Duration)

### Model 2 (MRM_2_)

General Equation (1991–1995)

$${\text{Y}=\alpha +\beta }_{1}{\text{X}}_{1}{+\beta }_{2}{\text{X}}_{2}{–\beta }_{3}{\text{X}}_{3}{+\beta }_{4}{\text{X}}_{4}{–\beta }_{5}{\text{X}}_{5}{+\beta }_{6}{\text{X}}_{6}{–\beta }_{7}{\text{X}}_{7}$$

BPD Outbreak(%)=129.9+0.03(Month)+0.005(State)-0.06(Year)+0.001(Rainfall)-0.03(Average Temperature)+0.005(Average Relative Humidity)-0.04(Sunshine Duration)

### Model 3 (MRM_3_)

General Equation (1991–1995)

$${\text{Y}=\alpha +\beta }_{1}{\text{X}}_{1}{–\beta }_{2}{\text{X}}_{2}{–\beta }_{3}{\text{X}}_{3}{+\beta }_{4}{\text{X}}_{4}{–\beta }_{5}{\text{X}}_{5}{+\beta }_{6}{\text{X}}_{6}$$

BPD Outbreak(%)=127.8+0.02(Month)-0.002(State)-0.06(Year)+0.001(Rainfall)-0.05(Average Temperature)+0.007(Average Relative Humidity)

### Model 4 (MRM_4_)

General Equation (1991–1995)

$${\text{Y}=\alpha –\beta }_{1}{\text{X}}_{1}{+\beta }_{2}{\text{X}}_{2}{–\beta }_{3}{\text{X}}_{3}{–\beta }_{4}{\text{X}}_{4}{–\beta }_{5}{\text{X}}_{5}{–\beta }_{6}{\text{X}}_{6}{+\beta }_{7}{\text{X}}_{7}{+\beta }_{8}{\text{X}}_{8}{–\beta }_{9}{\text{X}}_{9}$$

BPD Outbreak(%)=101–0.008(Month)+0.02(State)-0.05(Year)-0.002(Rainfall)-0.02(Max. Temperature)-0.06(Min. Temperature)+0.01(Relative Humidity-Morning)+0.01(Relative Humidity-Afternoon)-0.1(Sunshine Duration)

### Model 5 (MRM_5_)—ETAPOD

General Equation (1985–2014) **[Accepted Equation]**

$${\text{Y}=-\alpha -\beta }_{1}{\text{X}}_{1}{+\beta }_{2}{\text{X}}_{2}{+\beta }_{3}{\text{X}}_{3}$$

BPD Outbreak(%)=-20.4–0.004(Rainfall)+0.272(Relative Humidity)+0.511(Temperature)

### Model 6 (MRM_6_)

General Equation (1991–1995)

$${\text{Y}=\alpha +\beta }_{1}{\text{X}}_{1}{–\beta }_{2}{\text{X}}_{2}{+\beta }_{3}{\text{X}}_{3}{–\beta }_{4}{\text{X}}_{4}{–\beta }_{5}{\text{X}}_{5}{+\beta }_{6}{\text{X}}_{6}{–\beta }_{7}{\text{X}}_{7}$$

BPD Outbreak(%)=101.6–0.007(Month)+0.02(State)-0.05(Year)-0.002(Rainfall)-0.07(Average Temperature)+0.02(Average Relative Humidity)-0.1(Sunshine Duration)

### Model 7 (MRM_7_)

General Equation (1985–2014)

$${\text{Y}=-\alpha -\beta }_{1}{\text{X}}_{1}{+\beta }_{2}{\text{X}}_{2}{-\beta }_{3}{\text{X}}_{3}{+\beta }_{4}{\text{X}}_{4}$$

BPD Outbreak(%)=-1364–0.00099(Rainfall)+0.008(Relative Humidity)–1.38(Temperature)+0.705(Year)

### Model 8 (MRM_8_)

General Equation (1991–1995)

$${\text{Y}=-\alpha -\beta }_{1}{\text{X}}_{1}{+\beta }_{2}{\text{X}}_{2}{-\beta }_{3}{\text{X}}_{3}{+\beta }_{4}{\text{X}}_{4}$$

BPD Outbreak(%)=-1.64–0.00152(Rainfall)-0.0727(Average Temperature)+0.02(Average Relative Humidity)-0.119(Sunshine Duration)

### Model selection

**Fig 13:**
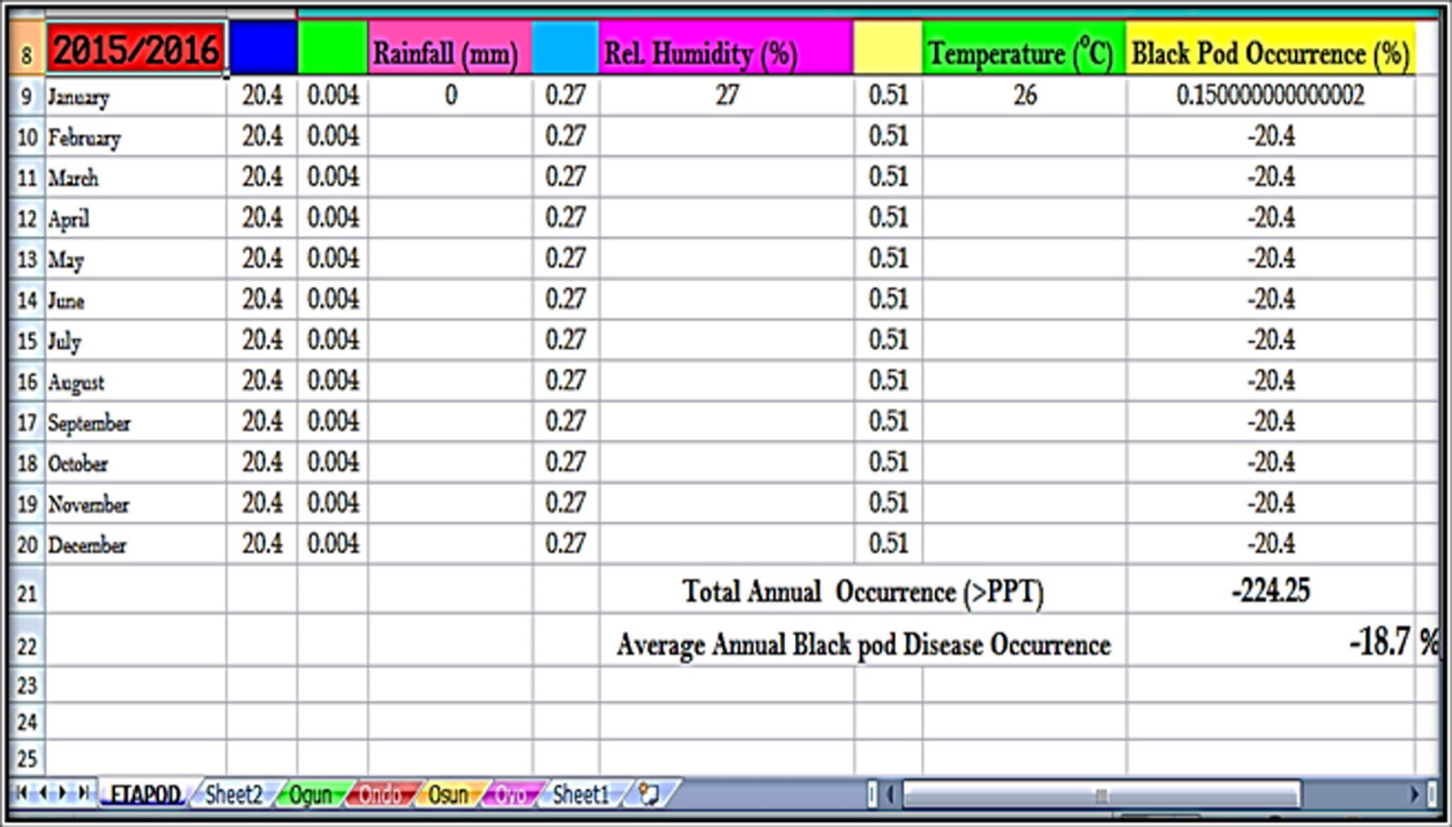
MRM_5_ BPD prediction model (ETAPOD).

The posthoc analysis conducted showed that MRM_5_ was the preferred model for BPD prediction followed by MRM_4_>MRM_1_>MRM_2_>MRM_3_ in terms of the Standard Error of Regression (SER) which was given as 0.22, 0.39, 0.45, 0.45, and 0.45 respectively; Root Mean Square Error of Prediction (RMSE_pred._): 0.30, 0.39, 0.46, 0.46 and 0.46 respectively; and the Adjusted Co-efficient of Correlation (R-Sq_Adj._): 0.67, 0.49, 0.32, 0.32 and 0.31 for MRM_5_, MRM_4_, MRM_1_, MRM_2_, and MRM_3_. The preferred model MRM_5_ was named “ETAPOD” (Fig 13)

### Prediction of annual BPD outbreak by ETAPOD and confirmation of forecast results

**Fig 14:**
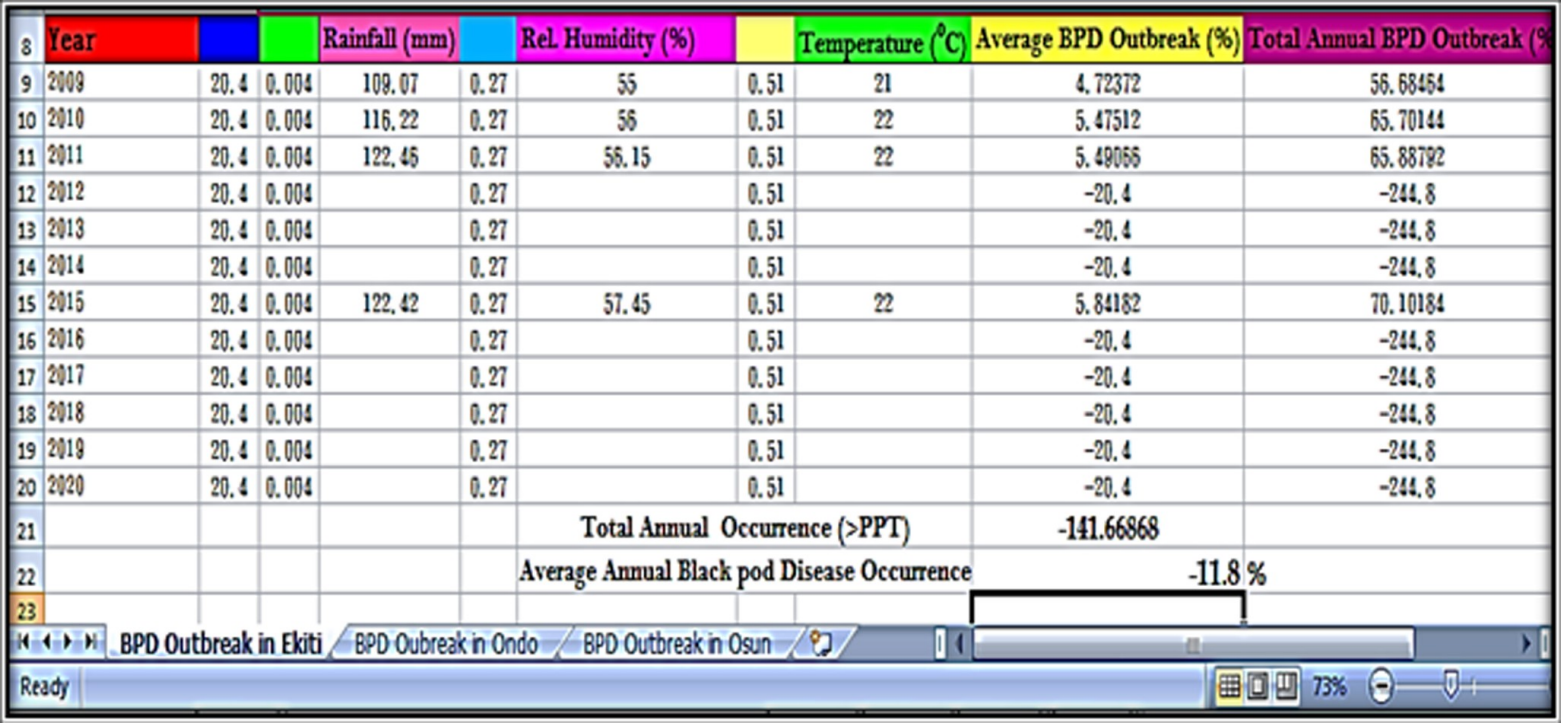
BPD outbreak predictions for Ekiti state (2009–2015).

**Fig 15:**
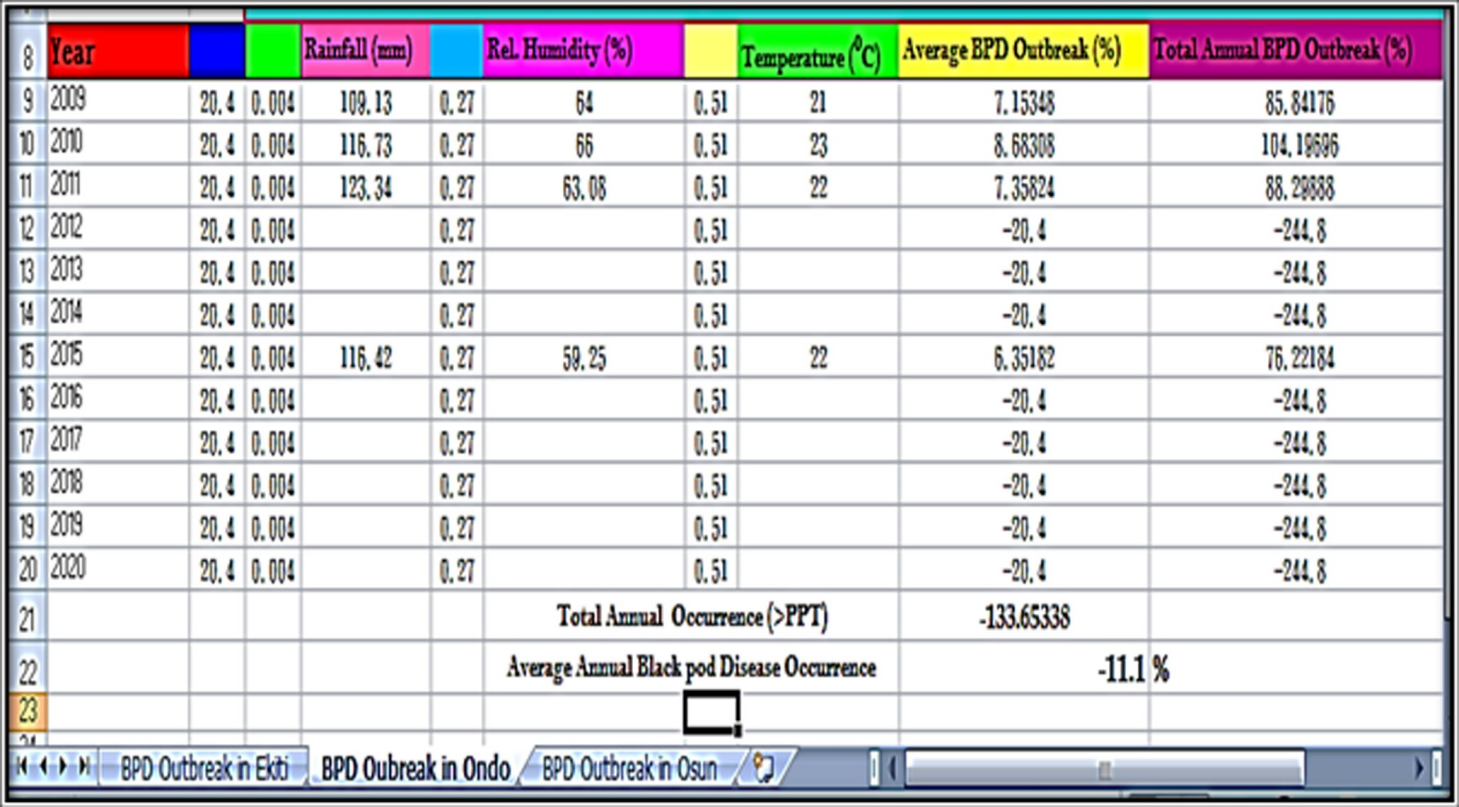
BPD outbreak predictions for Ondo state (2009–2015).

**Fig 16:**
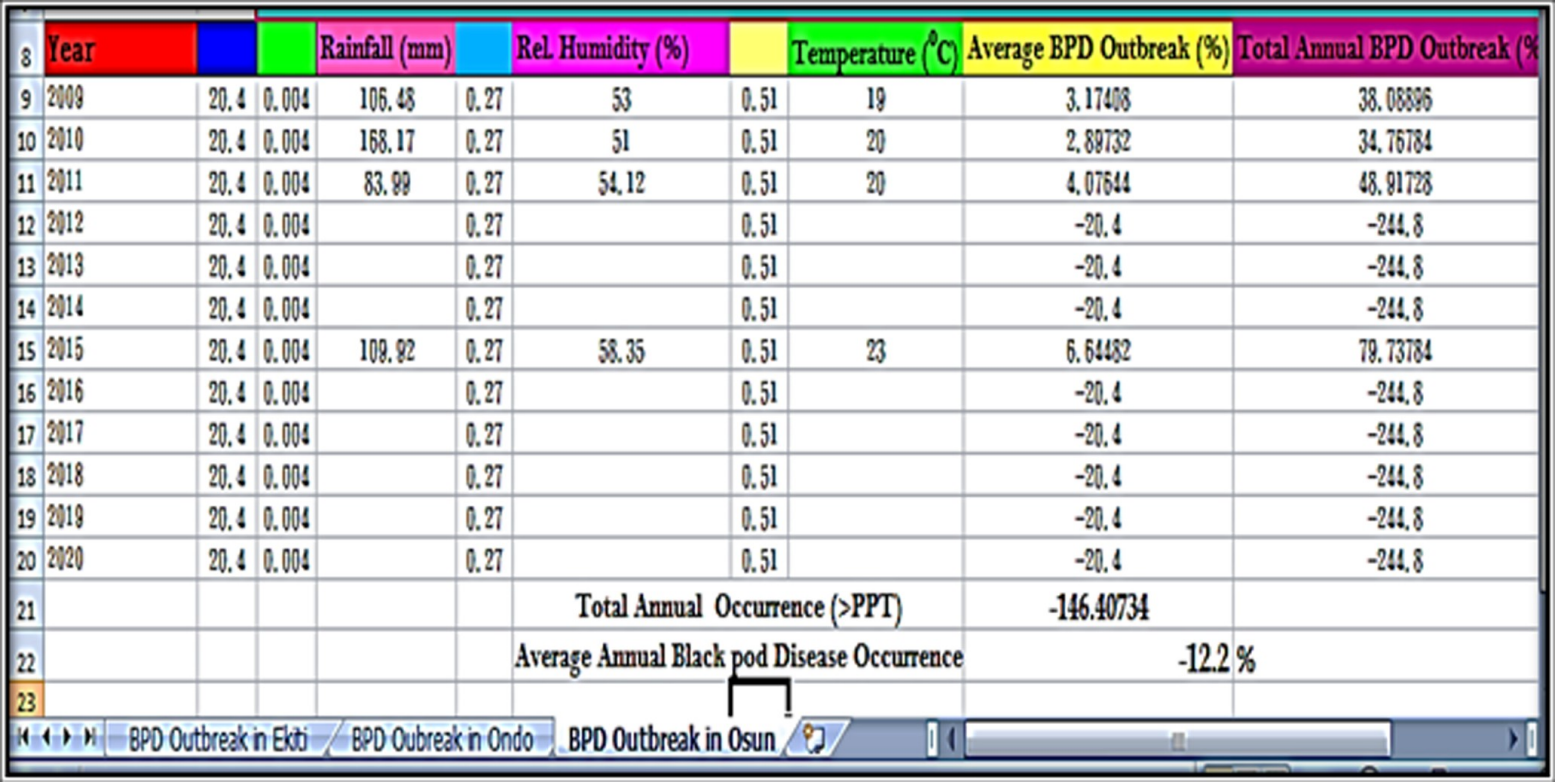
BPD outbreak predictions for Osun state (2009–2015).

The annual BPD outbreak for Ekiti, Ondo and Osun States (Southwest, Nigeria) were used to test the developed BPD forecast model (ETAPOD). In 2009, Ekiti, Ondo and Osun States had total annual BPD outbreak of 53.0, 71.0 and 5.0%, respectively (Table 8). The prediction was true for Ekiti and Ondo (56.7 and 85.9%, respectively) as stated in Figs 14–15, but BPD outbreak was inaccurately predicted for Osun State (38.1%) as stated in Fig 16. ETAPOD predicted inaccurately for Osun only among all the States in 2010. In 2011, the result for BPD outbreak was accurately predicted for Ekiti and Osun States (65.9 and 48.9%, respectively) compared to their actual values (71.0 and 69.0%, respectively), while that of Ondo State was under estimated by the developed model (Actual occurrence was 178% compared to the predicted value of 88.3% (Table 8). In 2015, the predicted results for BPD outbreak in all the States were a true reflection of the actual level of the disease outbreak observed within that period i.e. Ekiti (Actual = 67.0% and Predicted = 70.1%), Ondo (Actual = 63.1% and Predicted = 76.2%), and Osun (Actual = 55.2% and Predicted = 79.7%), respectively (Table 8).

**Table 8:** Annual BPD outbreak in Southwest, Nigeria compared with predictions from ETAPOD.

	Annual BPD outbreak (%)					
Year	Ekiti		Ondo		Osun	
	Actual (%)	Predicted (%)	Actual (%)	Predicted (%)	Actual (%)	Predicted (%)
2009	53.0	56.7	71.0	85.9	5.0	38.1
2010	69.0	65.7	126.0	104.2	11.0	34.8
2011	71.0	65.9	178.0	88.3	69.0	48.9
2015	67.0	70.1	63.1	76.2	55.2	79.7

### The level of performance of MRM_5_ forecast model (ETAPOD)

ETAPOD had 100% performance rating for BPD prediction in Ekiti (2009, 2010, 2011 and 2015) with model efficiency of 95–100%. The performance of the model was rated 80% in 2010 and 2015 (Ondo) with model efficiency of 85–90%, 70% in 2011 (Osun) with model efficiency of 81–84%, 60% in 2010 (Ondo and Osun) and 2015 (Osun) with model efficiency of 75–80%, 40% in 2009 (Osun) with model efficiency of 65–69% and 0% 1n 2011 (Ondo) with model efficiency between 0 and 49% (Table 9).

**Table 9:** The performance level of the developed BPD prediction model.

	Performance of ETAPOD [MRM_5_ Forecast Model] (%)		
Year	Ekiti	Ondo	Osun
2009	100.0	80.0	40.0
2010	100.0	60.0	60.0
2011	100.0	0.0	70.0
2015	100.0	80.0	60.0
**Note:**			
Difference in BPD outbreak		Performance Rating	Model Efficiency
0–5%		100%	95–100%
6–10%		90%	90–94%
11–15%		80%	85–89%
16–20%		70%	81–84%
21–25%		60%	75–80%
26–30%		50%	70–74%
31–35%		40%	65–69%
36–40%		30%	60–64%
41–45%		20%	55–59%
46–50%		10%	50–54%
Above 50%		0%	0–49%

### The overall assessment of the output quality of MRM_5_ forecast model (ETAPOD)

The quality of forecast result was very high in Ekiti (2009, 2010, 2011 and 2015), Ondo (2009, 2010 and 2015) and Osun (2009, 2010, 2011 and 2015), respectively. A very poor forecast quality was observed in Ondo State (2011) as stated in Table 10.

**Table 10:** Assessment of the quality of predictions made by ETAPOD.

	Forecast Quality of ETAPOD (%)		
Year	Ekiti	Ondo	Osun
2009	+++	+++	+++
2010	+++	+++	+++
2011	+++	-	+++
2015	+++	+++	+++

**“**+” Fairly Good Prediction, **“**++” Good Prediction, “+++” Extremely Good Prediction, and “-”Poor Prediction.

### The error in prediction of BPD outbreak by ETAPOD

The error for black pod disease outbreak prediction was very low in Ekiti State i.e. 13.7, 10.9, 26.0 and 9.6, respectively for 2009, 2010, 2011, and 2015 (Table 11). It was extreme for Ondo (8046.1) and Osun State (1095.6), respectively (Table 11).

**Table 11:** The error in prediction of BPD by MRM_5_ forecast model (ETAPOD).

	Prediction Error (%) = (Y-Ŷ)^2^		
Year	Ekiti	Ondo	Osun
2009	13.7	222.0	1095.6
2010	10.9	475.2	566.4
2011	26.0	8046.1	404.0
2015	9.6	171.6	600.3

### Accuracy of ETAPOD in BPD prediction

The model was very accurate in the prediction of BPD outbreak in Ekiti with precision i.e. 93, 95, 93 and 95%, respectively for 2009, 2010, 2011, and 2015. In Ondo, the accuracy level of BPD determination by ETAPOD was 79, 83, 50 and 79% for 2009, 2010, 2011, and 2015, respectively; whereas, in Osun the precision level was very low (0, 0, 71 and 56%, respectively for 2009, 2010, 2011, and 2015) (Table 12).

**Table 12:** Determination of the accuracy level of the developed prediction model for BPD.

	BPD Prediction Accuracy [100 - (/Y-Ŷ/)/Y x 100)]		
Year	Ekiti	Ondo	Osun
2009	93%	79%	0%
2010	95%	83%	0%
2011	93%	50%	71%
2015	95%	79%	56%

### The probability of obtaining accurate BPD predictions

The probability of obtaining accurate results for BPD prediction was very high in Ekiti and Ondo States, but it was not consistent in Osun State (Table 13). The probability range for obtaining good results in Ekiti was 0.93≤P≤0.95, whereas, it was 0.50≤P≤0.83 in Ondo State. In Osun State, the value was a disappointing 0.00≤P≤0.71 range (Table 13).

**Table 13:** The probability of obtaining good BPD outbreak predictions from ETAPOD.

Year	Ekiti		Ondo		Osun	
	Error Level	Prob. Level	Error Level	Prob. Level	Error Level	Prob. Level
2009	0.07	0.93	0.21	0.79	1.00	0.00
2010	0.05	0.95	0.17	0.83	1.00	0.00
2011	0.07	0.93	0.50	0.50	0.29	0.71
2015	0.05	0.95	0.21	0.79	0.44	0.56

## Discussion

### Weather survey in line with BPD outbreak in Southwestern Nigeria

The weather report in the early 1900s for Southwestern Nigeria showed that there was recurrent rainfall within the months of March through October from 1991 to 1995. Also, ambient temperature was low during the day and at night, and there was much saturated water vapour in the air across the four (4) States investigated within the same period. March to October happen to be the most productive periods for Cocoa production in Southwest, Nigeria; Therefore, the observations noted gives an indication of the possibility of infection within these periods. This favourable weather pattern for black pod disease infection was earlier reported by Akrofi [14].

### BPD prediction and data validation

The MRM_5_ BPD forecast model (ETAPOD) selected as the best fitted model for prediction of BPD outbreak in Nigeria gave results of annual BPD outbreak that accurately quantified annual BPD outbreak in Ekiti and Ondo States but inaccurately described the situation in Osun State. This is as a result of the credibility of the data fed into the model. The observation made was in accordance with the findings of Luo [15] who also designed a forecast model for the prediction of foliar diseases of winter wheat caused by *Septoria tritici* across England and Wales and his predictions for the disease was seemingly not 100% accurate.

The error of BPD prediction was very low in Ekiti, whereas, it was on the high side in Osun. The disparity in the credibility of the predicted outcome is solely due to the quality of the data fed into the system. This lapses was indeed identified by Luo [15] who gave a few recommendations on how a forecast system can be improved in order to obtain quality forecast results. The level of prediction accuracy was defined thus as 0.0%≤Accuracy Level<100%. This was also identified by Luo [15] as he recognized the fact that no forecast system can be 100% accurate at all times and in all instances.

### Recommendation

ETAPOD harnesses several potentials and possibilities that can be improved on to obtain excellent results. The accuracy of the warning system developed for the prediction of black pod disease (ETAPOD) can be perfected if:

1. Weather parameters are obtained from meteorological stations situated in the farm
2. Consistency of cocoa production within that locality is constant
3. The type of cropping system employed could be determined
4. cocoa is the major crop cultivated on the piece of land
5. Advanced digital image analysis could be used to improve measurement precision of disease prevalence and severity.

## Conclusion

ETAPOD harnesses the potentials to improve the functionality of other existing management strategies for the control of BPD in Nigeria by providing timely information on its outbreak, detect areas under severe attack (AUSA), thereby discouraging fungicide misuse among local cocoa farmers. ETAPOD is unique in the sense that its primary function is not geographically restricted. Also, ETAPOD can be manipulated to provide optimum results anywhere needed in Nigeria, Africa and all around the world. Its ability to provide qualitative and quantitative description of BPD pressure makes it superior to other forms of BPD control strategies in use. Therefore, ETAPOD is a pertinent tool that can effectively minimize the prevalence of BPD in Nigeria with minimal chemical application, decreasing the risk of chemical poisoning and increasing the production of healthy cocoa products nationwide. This is the surest and fastest way to ensure sustainability of cocoa production in Nigeria and the world at large.

## Supporting information

S1 Fig Hypothetical review of the effect of pathogen’s inoculum load on BPD development. (TIF)

S2 Fig Theoretical establishment of the effect of rainfall on BPD development. (TIF)

S3 Fig A putative description of temperature effects on BPD development. (TIF)

S4 Fig Hypothetical representation of humidity and BPD development. (TIF)

S5 Fig A proposed relationship between sunlight duration and BPD development. (TIF)

S6 Fig A conjectural examination of the effects of wind speed on BPD development. (TIF)

S7 Fig A review of the effects of timing and how it affects BPD development. (TIF)

S8 Fig Atmospheric pressure and its correlation with BPD development. (TIF)

S1 Dataset Doctoral research data. (XLSX)

S2 Dataset BPD data for model validation and optimization. (XLSX)
