# Supplementary figures and images for: ETAPOD: A forecast model for prediction of black pod disease outbreak in Nigeria
Source: PLoS One. 2020 Jan 10;15(1):e0209306. doi: 10.1371/journal.pone.0209306 (PMC6953891; doi:10.1371/journal.pone.0209306)

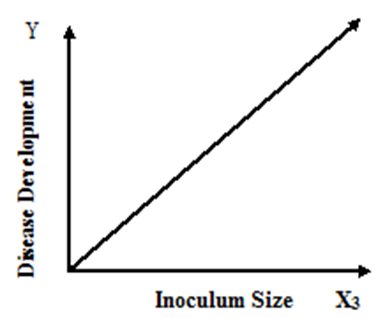

Supplement: S1 Fig — (TIF) [file pone.0209306.s001.tif]

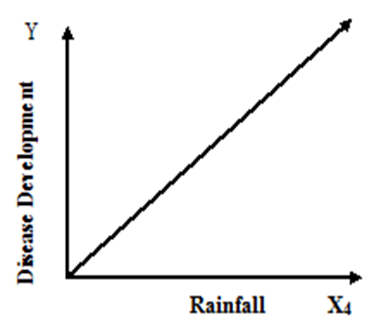

Supplement: S2 Fig — (TIF) [file pone.0209306.s002.tif]

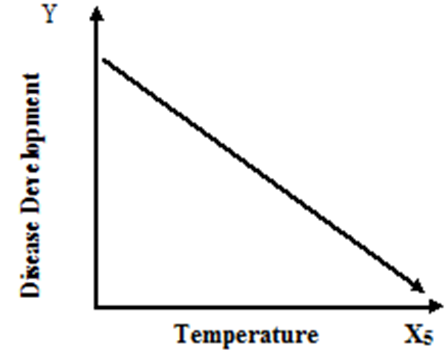

Supplement: S3 Fig — (TIF) [file pone.0209306.s003.tif]

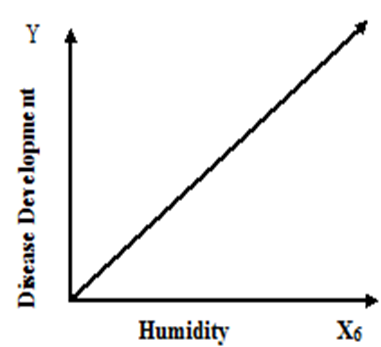

Supplement: S4 Fig — (TIF) [file pone.0209306.s004.tif]

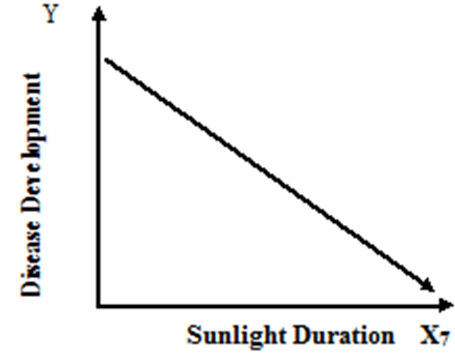

Supplement: S5 Fig — (TIF) [file pone.0209306.s005.tif]

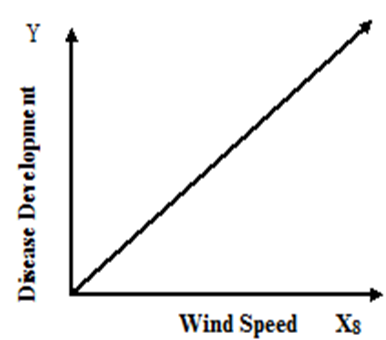

Supplement: S6 Fig — (TIF) [file pone.0209306.s006.tif]

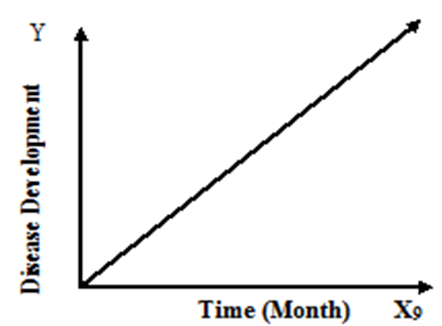

Supplement: S7 Fig — (TIF) [file pone.0209306.s007.tif]

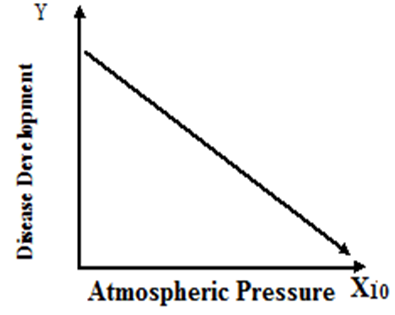

Supplement: S8 Fig — (TIF) [file pone.0209306.s008.tif]
